# Une pemphigoïde bulleuse localisée radio-induite

**DOI:** 10.11604/pamj.2013.14.43.2358

**Published:** 2013-01-30

**Authors:** Fadwa El Amrani, Badredine Hassam

**Affiliations:** 1 Service de dermatologie, CHU Ibn Sina, université Med V, Souissi, Rabat, Maroc

**Keywords:** Pemphigoïde bulleuse, complication, radiothérapie, bullous pemphigoid, complication, radiotherapy

## Image en médicine

La pemphigoïde bulleuse (PB) radio-induite est une complication rare de la radiothérapie. Elle a principalement été décrite chez des femmes traitées pour un cancer du sein. Elle se caractérise par la survenue, au cours de la radiothérapie ou dans l'année qui la suit, de lésions bulleuses tendues et prurigineuses, généralement au niveau du site de l'irradiation. Sa pathogénie reste inconnue. Son pronostic est souvent bon avec une résolution sans séquelles sous dermocorticoïdes. Nous rapportons le cas d'une patiente de 67 ans, ayant comme antécédent une mastectomie droite avec curage ganglionnaire suivis de chimio-radiothérapie pour un carcinome canalaire infiltrant du sein. Un mois après l'arrêt de la radiothérapie, sont apparues des lésions bulleuses tendues et prurigineuses, à contenu séro-hématique, strictement localisées au site d'irradiation. La biopsie cutanée a montré l'aspect de bulle sous épidermique et l'immunofluorescence directe un dépôt linéaire d'IgG et de C3 le long de la membrane basale, ce qui a soulevé le diagnostic de PB. Un traitement local à base de propionate de clobétasol (Dermoval^®^) a été entrepris. L’évolution était favorable avec disparition des lésions sans séquelles.

**Figure 1:**
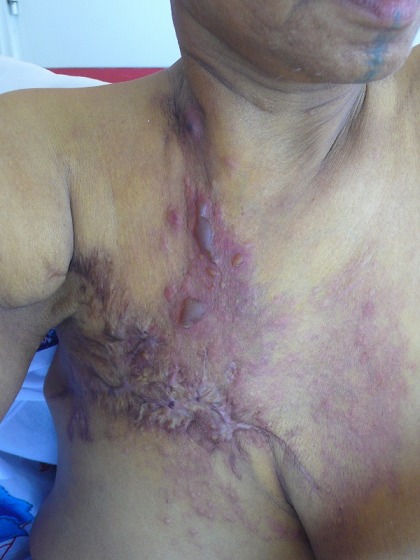
Lésions bulleuses tendues et prurigineuses, à contenu séro-hématique, strictement localisées au site d'irradiation chez une patiente de 67 ans, ayant comme antécédent une mastectomie droite avec curage ganglionnaire suivis de chimio-radiothérapie pour un carcinome canalaire infiltrant du sein.

